# Circulating Endothelial Microparticles and Correlation of Serum 1,25-Dihydroxyvitamin D with Adiponectin, Nonesterified Fatty Acids, and Glycerol from Middle-Aged Men in China

**DOI:** 10.1155/2016/8420768

**Published:** 2016-05-25

**Authors:** Zhongxiao Wan, Lugang Yu, Jinbo Cheng, Zengli Zhang, Baohui Xu, Xing Pang, Hui Zhou, Ting Lei

**Affiliations:** ^1^Department of Nutrition and Food Hygiene, School of Public Health, Soochow University, 199 Renai Road, Suzhou 215123, China; ^2^Jiangsu Key Laboratory of Preventive and Translational Medicine for Geriatric Disease, Soochow University, 199 Renai Road, Suzhou 215123, China; ^3^Suzhou Industrial Park center Disease Control & Prevention, 58 Suqian Road, Suzhou 215123, China; ^4^Department of Labor Hygiene and Environmental Health, School of Public Health, Soochow University, 199 Renai Road, Suzhou 215123, China

## Abstract

The aim of the present study is (1) to determine the correlation between circulating 1,25-dihydroxyvitamin D [25(OH)D] and adiponectin, nonesterified fatty acids (NEFAs), and glycerol and (2) to determine the alterations in circulating endothelial microparticles (EMPs) in Chinese male subjects with increased body mass index (BMI). A total of 45 male adults were enrolled with varied BMI [i.e., lean, overweight (OW), and obese (OB), *N* = 15 per group]. Blood samples were collected under overnight fasting condition, and plasma was isolated for the measurement of endothelial microparticles (EMPs), total and high-molecular weight (HMW) adiponectin, 25(OH)D, nonesterified fatty acids (NEFAs), and glycerol. Circulating 25(OH)D levels were inversely associated with total adiponectin, NEFA, and glycerol levels. There is no difference for CD62E+ or CD31+/CD42b− EMPs among 3 groups. In Chinese male adults with varied BMI, an inverse correlation existed between 25(OH)D levels and total adiponectin, NEFA, and glycerol levels; and there is no significant difference for CD62E+ or CD31+/CD42b− EMPs among lean, overweight, and obese subjects.

## 1. Introduction

Obesity is one of the main causal factors for enhancing the morbidity and mortality rates of metabolic chronic diseases such as type 2 diabetes (T2DM) and cardiovascular diseases (CVDs) worldwide [1]. The considerable role of adipose tissue in these complications is well recognized. This is largely owing to the fact that adipose tissue secretes a wide range of biologically active adipokines and cytokines with modulatory effects on glucose homeostasis and lipid metabolism [2]. Among these, adiponectin is one of the most abundant adipokines secreted from adipose tissue [3] and circulating adiponectin is inversely related to adiposity [4]. Circulating adiponectin is composed of trimer, hexamer, and high-molecular weight (HMW) forms [5]. HMW form is considered as the major active form of adiponectin and a better marker for insulin resistance and metabolic syndrome [6]. Another main function of adipose tissue is providing fuel for the body under energy demanding condition via lipolysis, which will result in elevation in circulating glycerol and FFAs level [7, 8]. Vitamin D deficiency is also prevalent in obese subjects [9] and serum 1,25-dihydroxyvitamin D [25(OH)D] is inversely associated with BMI [10]. Existing evidence also suggested an association between circulating 25(OH)D and adiponectin levels. For example, via the Mendelian randomization approach, Husemoen et al. [11] speculated that a possible causal association existed between serum 25(OH)D and total adiponectin, while further studies are required to confirm this. Consequently, more studies are required to explore the associations between vitamin D and adiponectin (total and HMW form), NEFAs, and glycerol.

Endothelial microparticles (EMPs) are complex vesicular structures shed from endothelial cells in response to stimuli such as inflammatory activation and/or apoptosis [12]. They are now considered as novel biomarkers of endothelial activation and damage that are increased in overweight/obese individuals at risk for metabolic syndrome (MetS) [13, 14]. It remains unknown whether EMPs will be altered in the transition from lean to obese status from Chinese subjects.

With the above points in mind, we thought it is important (1) to determine the correlation between vitamin D and adiponectin, NEFAs, and glycerol and (2) to determine the alterations in circulating EMPs in Chinese male subjects with increased BMI. This will help us understand the complex biology of obesity in adipose tissue and its roles in affecting circulating markers (such as vitamin D and EMPs). We hypothesized that (1) positive correlations existed between vitamin D and adiponectin (total and HMW form), while negative correlations existed between vitamin D and NEFAs and glycerol, and that (2) EMPs might be altered under obese condition.

## 2. Materials and Methods

### 2.1. Study Subjects

From October to December 2014, we enrolled 45 male adults (aged 45–60 years) with no history of cardiovascular disease or type 2 diabetes from Suzhou Industrial Park area, Suzhou, China. Among these, there are 15 subjects in lean (LN), overweight (OW), and obese (OB) group, respectively. According to the working group on obesity in China (WGOC) [15], lean was defined as 18.5 ≤ BMI ≤ 23.9 kg/m^2^; OW was defined as 24.0 ≤ BMI ≤ 27.9 kg/m^2^; and OB was defined as BMI ≥ 28 kg/m^2^. All subjects recruited did not have metabolic syndrome or hypertension, while subjects in the obese group have dyslipidemia characterized by elevated triglyceride. The present study was conducted according to the guidelines laid down in the Declaration of Helsinki, and all procedures involving human subjects were approved by the Human Research and Ethical Committee of the Soochow University and all participants provided signed informed consent.

### 2.2. Biological Sampling and Measurement

Subjects reported to the laboratory after an overnight fast at 8 am. Blood samples (5 mL) were obtained by venipuncture from an antecubital vein and collected into EDTA tubes. Blood was centrifuged at 1500 g for 10 mins at 4°C and plasma immediately frozen at −80°C for subsequent analyses. Plasma 25(OH)D was analyzed using 25 OH vitamin D reagent based on the chemiluminescent immunoassay (CLIA). Total and HMW adiponectin were measured via ELISA kit from ALPCO Immunoassays (cat#47-ADPMS-E01). Plasma glycerol and NEFAs were measured by glycerol assay kit (cat#E1002, Applygen Technologies, Beijing, China) and Labassay NEFA kit (cat#294-63601, Wako, Osaka, Japan), respectively. The assays for adiponectin, HMW adiponectin, glycerol, and NEFAs were run in duplicate; the average CV for duplicates is <10% in our laboratory.

### 2.3. Endothelial Microparticles (EMPs) Measurement

Circulating EMPs were measured in platelet poor plasma by flow cytometry following the methods of Jenkins et al. [16]. In brief, frozen plasma samples were thawed at room temperature for 20 minutes and centrifuged at 1500 g for 15 minutes. The top two-thirds of plasma were then further centrifuged at 1500 g for another 15 minutes to obtain platelet poor plasma. The top 100 *μ*L of platelet poor plasma was then incubated with fluorochrome labeled antibodies specific for PE-CD42b, FITC-CD31, and APC-CD62E for 20 minutes in the dark at 4°C. Samples were then fixed with 93 *μ*L of 2% paraformaldehyde and diluted up to 500 *μ*L with sterile, 0.2 *μ*M filtered, PBS and analyzed on a FC500 Beckman Coulter (CA, USA). A microparticle size gate was determined using 900 nm Latex beads which is carboxylate-modified polystyrene. Unstained and fluorescence minus one controls were used to differentiate between true events and background/debris. EMPs were identified as CD62E+ and CD31+/CD42b− events within the microparticle size gate.

### 2.4. Statistical Analysis

All data are presented as mean ± SEM. Statistical analyses were performed using SPSS 20.0 (SPSS Inc., Chicago, IL, USA). Comparisons among groups were made using a one-way ANOVA followed by Tukey's post hoc test. Relation between serum 25(OH)D level and other indices was analyzed by Pearson's correlation analysis. Statistical significance was set at *p* < 0.05.

## 3. Results

### 3.1. Circulating 25(OH)D, Total and HMW Adiponectin, NEFAs, and Glycerol Level

As illustrated in Table 1, plasma levels of glycerol were significantly higher in the OW and OB group compared to lean group. Meanwhile, serum NEFAs were significantly increased in the OB group compared to lean group. In contrast, compared to the lean group, HMW adiponectin was decreased in the OW and OB group; and total adiponectin was decreased in the OB group.

### 3.2. Relationship between 25(OH)D Level and Other Metabolic Parameters

As shown in Table 2, 25(OH)D levels were inversely associated with NEFAs and glycerol levels and positively associated with total adiponectin. There were no significant correlations between 25(OH)D level and HMW adiponectin.

### 3.3. Circulating Endothelial Microparticles from the Subjects

**Figure 1:**
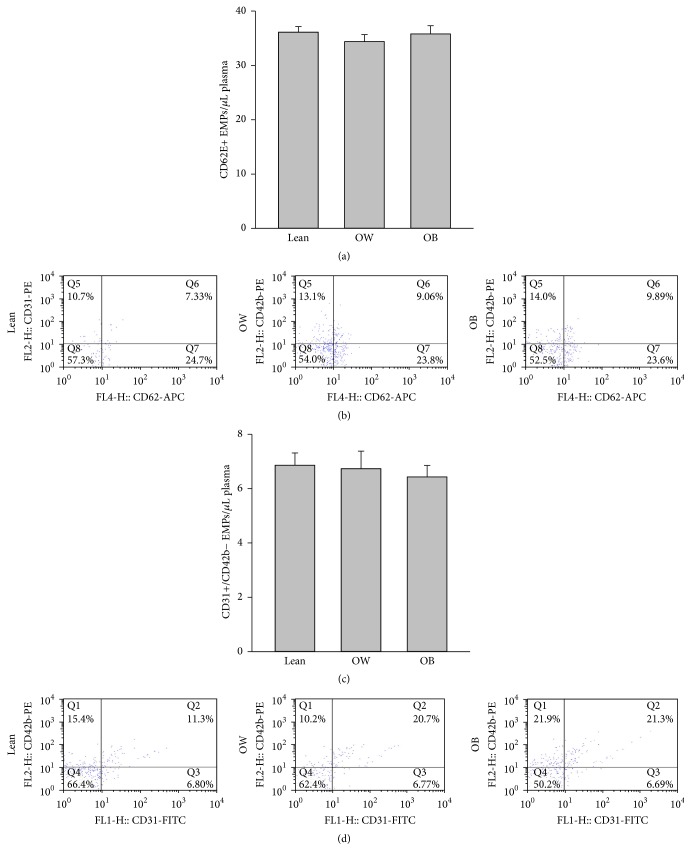
Circulating CD62E+ and CD31+/CD42b− EMPs levels among groups. Circulating EMPs were measured in platelet poor plasma by flow cytometry with fluorochrome labeled antibodies specific for PE-CD42b, FITC-CD31, and APC-CD62E. EMPs were identified as CD62E+ and CD31+/CD42b− events with a diameter <1 *μ*M. There is no difference for circulating CD62E+ (a) and CD31+/CD42b− EMPs (c) among the 3 groups. Representative fluorescence-activated cell sorter dot plots of CD62E+ and CD31+/CD42b− EMPs from lean, overweight, and obese group were shown in (b) and (d), respectively. Data are presented as mean ± SEM (*N* = 15).

As shown in Figure 1, there is no difference for CD62E+ or CD31+/CD42b− EMPs between lean, overweight, and obese group.

## 4. Discussion

Serum 25(OH)D is inversely associated with BMI [10]. Previously, Husemoen et al. [11] suggested that a possible causal association existed between serum 25(OH)D and total adiponectin, and there was no association between serum 25(OH)D and HMW adiponectin. Adiponectin is synthesized as a single subunit which undergoes multimerisation to form HMW multimers prior to secretion [5]. We are perplexed by the lack of association between serum 25(OH)D and HMW adiponectin considering the close association between total and HMW adiponectin [17]. We think several reasons might explain this phenomenon. First, Schraw et al. [5] demonstrated that, for female subjects, on average half of their adiponectin is in the HMW form with the other half being hexamer and trimer, while, for male subjects, approximately equal levels of all three complexes existed in circulation. Because our subjects are solely males, HMW form could not represent the total adiponectin; accordingly a dissociation might exist for the correlations between total, HMW form adiponectin, and 25(OH)D. Second, efficient multimerisation to form the HMW multimers depends on multiple posttranslational modifications and is very sensitive to physiological stimuli such as high glucose [18]. It is likely that the posttranslational modifications of adiponectin and physiological factors such as glucose might explain the lack of the association between 25(OH)D and HMW multimers. Nevertheless, further studies by utilizing approaches that could dynamically detect the different forms of adiponectin such as mass spectrometry and gel filtration chromatography are required to further explore the associations between serum 25(OH)D and different forms of adiponectin.

Our study confirmed this finding in Chinese male subjects with varied BMI. Meanwhile, we found an inverse association existing between 25(OH)D levels and NEFA and glycerol levels. Our study is the very first to report a negative association between 25(OH)D levels and NEFAs, as well as glycerol. Vitamin D has been reported to affect adipose tissue function such as inhibiting lipolysis in human adipocytes [19]; this might be one of the mechanisms for explaining the inverse association between 25(OH)D levels and NEFAs, as well as glycerol.

Elevation of EMPs has been rapidly accepted as an alternate surrogate marker of CVDs and endothelial function [20]. CD62E+ EMPs generally reflect endothelial activation or inflammation whereas CD31+/CD42b− EMPs are released upon endothelial cell apoptosis [12]. Circulating EMPs are increased in overweight/obese individuals with metabolic syndrome [21] and in various CVDs [22]. However, in our present study we found no difference for CD62E+ EMPs or CD31+/CD42b− EMPs between lean, overweight, and obese subjects. There are several reasons for explaining this. First, it is possible that the severity of obesity (or other risk factors such as MetS or CVDs) is required to elevate circulating EMPs. Our subjects are only obese; thus there is a lack of elevation in EMPs in our present study. Second, we only measured circulating CD62E+ and CD31+/CD42b− EMPs; it is possible that other EMPs' population such as CD146+ or CD41+ EMPs might be elevated. Last but not least, the genetic differences might also be one factor for affecting circulating EMPs. Regardless, our present study could suggest that, at least in Chinese male subjects, there is no alteration in CD62E+ and CD31+/CD42b− EMPs between lean, overweight, and obese subjects.

## 5. Limitations

Our study has several limitations. First, we only investigated Chinese male adults with a relatively small sample size; the extrapolation of the results to other populations will be limited. Second, we utilized Pearson's correlation analysis to explore the correlation between plasma vitamin D, total and HMW adiponectin, and other metabolic markers. There might be confounding factors for affecting the correlation analysis.

## 6. Conclusions

In summary, our present results showed that (1) in Chinese male adults with varied BMI, an inverse correlation existed between 25(OH)D levels and NEFA and glycerol levels, as well as positive association between 25(OH)D and total adiponectin levels and (2) there is no significant difference for CD62E+ or CD31+/CD42b− EMPs among lean, overweight, and obese subjects.

## Figures and Tables

**Table 1:** Clinical and biochemical parameter of subjects from different groups.

Variables	Lean	Overweight	Obese
Total adiponectin (ng/mL)	4.84 ± 0.47	4.14 ± 0.47	3.38 ± 0.52^*∗*^
HMW adiponectin (ng/mL)	1.76 ± 0.3	1.19 ± 0.25^*∗*^	1.09 ± 0.29^*∗*^
Vitamin D (ng/mL)	30.66 ± 1.83	27.81 ± 1.95	24.72 ± 1.58^*∗*^
NEFA (mM/L)	0.21 ± 0.03	0.20 ± 0.02	0.30 ± 0.04^*∗*^
Glycerol (mM/L)	26.26 ± 4.65	52.28 ± 10.23^*∗*^	58.82 ± 9.68^*∗*^

Data are expressed as mean ± SEM. ^*∗*^ *p* < 0.05 compared with the lean group.

**Table 2:** Correlation between 25(OH)D level and clinical parameters.

Variable	*r*	*p*
Total adiponectin (ng/mL)	0.306	0.048
HMW adiponectin (ng/mL)	0.07	0.724
NEFA (mM/L)	−0.573	0.01
Glycerol (mM/L)	−0.54	0.02

Data are expressed as mean ± SEM. Relation between serum 25(OH)D level and other indices was analyzed by Pearson's correlation analysis.
